# Molecular Pathogenesis of Colorectal Cancer with an Emphasis on Recent Advances in Biomarkers, as Well as Nanotechnology-Based Diagnostic and Therapeutic Approaches

**DOI:** 10.3390/nano12010169

**Published:** 2022-01-04

**Authors:** Fakhria A. Al-Joufi, Aseem Setia, Mounir M. Salem-Bekhit, Ram Kumar Sahu, Fulwah Y. Alqahtani, Retno Widyowati, Fadilah Sfouq Aleanizy

**Affiliations:** 1 Department of Pharmacology, College of Pharmacy, Jouf University, Aljouf 72341, Saudi Arabia; faaljoufi@ju.edu.sa; 2 Department of Pharmacy, Shri Rawatpura Sarkar University, Raipur 492015, India; 3 Department of Pharmaceutics, College of Pharmacy, King Saud University, Riyadh 11451, Saudi Arabia; fyalqahtani@ksu.edu.sa (F.Y.A.); Faleanizy@ksu.edu.sa (F.S.A.); 4 Department of Microbiology and Immunology, Faculty of Pharmacy, Al-Azhar University, Cairo 11884, Egypt; 5 Department of Pharmaceutical Science, Assam University (A Central University), Silchar 788011, India; 6 Department of Pharmaceutical Science, Faculty of Pharmacy, Universitas Airlangga, Surabaya 60115, Indonesia; rr-retno-w@ff.unair.ac.id

**Keywords:** colorectal cancer, biomarkers, cytotoxic medication, nanotechnology, genetic pathways, epigenetic changes, prognostic, phytomedicine

## Abstract

Colorectal cancer (CRC) is a serious disease that affects millions of people throughout the world, despite considerable advances in therapy. The formation of colorectal adenomas and invasive adenocarcinomas is the consequence of a succession of genetic and epigenetic changes in the normal colonic epithelium. Genetic and epigenetic processes associated with the onset, development, and metastasis of sporadic CRC have been studied in depth, resulting in identifying biomarkers that might be used to predict behaviour and prognosis beyond staging and influence therapeutic options. A novel biomarker, or a group of biomarkers, must be discovered in order to build an accurate and clinically useful test that may be used as an alternative to conventional methods for the early detection of CRC and to identify prospective new therapeutic intervention targets. To minimise the mortality burden of colorectal cancer, new screening methods with higher accuracy and nano-based diagnostic precision are needed. Cytotoxic medication has negative side effects and is restricted by medication resistance. One of the most promising cancer treatment techniques is the use of nano-based carrier system as a medication delivery mechanism. To deliver cytotoxic medicines, targeted nanoparticles might take advantage of differently expressed molecules on the surface of cancer cells. The use of different compounds as ligands on the surface of nanoparticles to interact with cancer cells, enabling the efficient delivery of antitumor medicines. Formulations based on nanoparticles might aid in early cancer diagnosis and help to overcome the limitations of traditional treatments, including low water solubility, nonspecific biodistribution, and restricted bioavailability. This article addresses about the molecular pathogenesis of CRC and highlights about biomarkers. It also provides conceptual knowledge of nanotechnology-based diagnostic techniques and therapeutic approaches for malignant colorectal cancer.

## 1. Introduction

Colorectal cancer (CRC) is a serious health issue in developed and developing countries alike, with the third highest incidence rate of all tumour-causing diseases [1]. This kind of cancer is a leading cause of morbidity and mortality in Western nations. Only a small percentage of CRC instances are caused by basic genetic abnormalities, with most cases occurring due to specific regular factors and growing age [2]. If left untreated, it can spread to the intestinal wall and the muscles beneath the surface of the skin. Environmental and genetic variables can also combine in many ways to accelerate cancer development and make it more difficult to treat.

In most cases, patients appear beyond the age of 60, with precursor starting adenomas that develop into cancer over for 1 to 2 decades [3]. Generalised screening of this population at age 50 years and older has been shown to reduce death from the disease, detect cancer at an earlier stage, and reduce the incidence of the disease. There have been four major hypotheses put out on the aetiology of colorectal cancer. To begin, the same genetic and epigenetic changes that cause colon cancer also increase the risk of developing colorectal cancer. Second, cancer develops in a staged manner, beginning with a precancerous condition and progressing to malignancy. Third, cancer development necessitates a lack of genetic stability. Fourth, in hereditary cancer syndromes, the germline forms of significant genetic abnormalities are generally associated with the development of rare colon cancers, which are caused by somatic occurrences of hereditary diseases [4].

Epigenetics, defined as heritable changes in gene expression without permanent changes in DNA sequence, plays an important role in the aetiology of different malignancies, including colorectal cancer. Recent epigenetic research has found a missing connection between certain gene expression patterns associated with CRC and the lack of genetic abnormalities. This missing link has now been discovered during the last two decades [5]. As an example, microsatellite in-stability (MSI), which is a hallmark of a molecular subgroup of CRC, results from an inability to repair DNA mismatches (MMRs) [6] properly. A genetic mutation can cause this defect in one of the MMR genes, the hypermethylation of the MLH1 gene’s promoter, or both. Chromosome instability (CIN) in CRC has also been linked to global hypomethylation [7]. There is further evidence that cancer-related pathways at the post-transcriptional level are influenced by microRNAs (miRNAs), which play a role in nearly all phases of CRC, from initiation through progression and metastasis. Cell development is inhibited by miR-143, which downregulates in CRC because it tar-gets the KRAS mRNA transcript [8]. Aside from improving our knowledge of CRC pathogenesis, these discoveries have also provided new opportunities to find disease biomarkers and treatment targets. The various CRC biomarkers such as BRAF mutations, NRAS, and KRAS, microsatellite instability (MSI), DNA mismatch repair (MMR) status, and CpG island methylation have been studied. A KRAS or NRAS mutation is associated with a worse prognosis, and anti-epidermal growth factor receptor (anti-EGFR) antibody treatment will be ineffective [9]. Mutation in BRAF V600E is seen in 8–10% of individuals with CRC. These patients have a more aggressive illness and a worse prognosis in the adjuvant and metastatic context. People with BRAF mutation and microsatellite instability-high (MSI-H) tumours had a better overall prognosis than people with BRAF mutation and microsatellite stable (MSS) illness, and it turns out, using MSI to screen for Lynch syndrome and identify patients who could benefit from immunotherapy is becoming increasingly important in CRC [10].

A variety of drug-loaded nanoparticles in the 20–400-nm size range have made significant contributions to chemotherapeutic drug delivery in recent years (e.g., liposomes, dendrimers, polymeric nanoparticles, and micelles) [11]. Many potential nanomedicine advancements are based on these systems, which have evolved from basic drug-loaded nanoparticles to multifunctional nanoparticles that target particular cancer cells by attaching them to specific cell-surface proteins. Antigens such as integrin and folic acid receptors, which are differentially expressed on the surface of cancer cells, can be targeted using targeted nanoparticles [12]. Several of these nanoparticles are now in clinical trials. A great deal has been learned about using nanoparticles as therapeutic platforms for treating malignancies ranging from the prostate to the ovary. The clinical development of nanoparticles for cancer therapy is restricted, despite the high death and morbidity rates associated with CRC. In this review, we emphasise in detail the pathogenesis of colorectal cancer and their suitable candidate as biomarkers and nanotechnology-based diagnostic methods and therapeutic approaches for malignant colorectal cancer. Additionally, it provided an outlook on therapeutic nanoparticles for the management of CRC.

## 2. Pathogenesis of Colorectal Cancer

**Figure 1:**
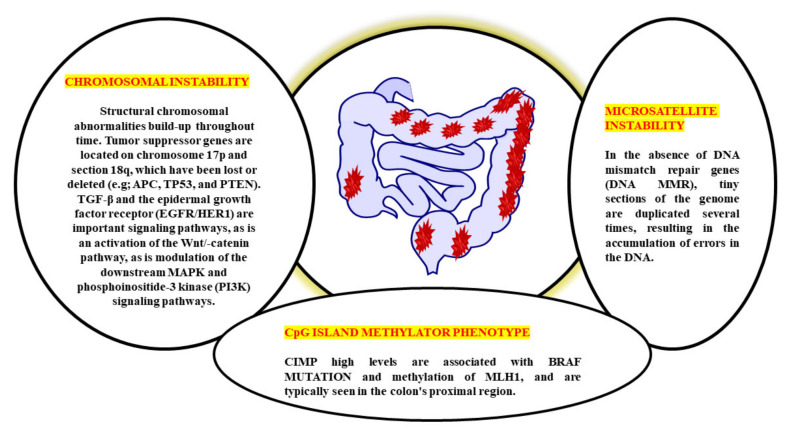
Multiple genetic pathways of CRC pathogenesis.

Genetic alterations in oncogenes and tumour suppressor genes occur sequentially from normal to the dysplastic epithelium in the adenoma–carcinoma process. This results in the development of CRC. Genomic changes, both genetic and epigenetic, convert normal glandular epithelium into adenocarcinoma when they accumulate [13]. While molecular mechanisms behind the cancer’s unabated development and spread remain a mystery, several genetic pathways have been proposed to explain CRC aetiology (Figure 1). CRC involves numerous molecular pathways, including the three major pathways: CIN, MSI, and CIMP, all of which play a crucial role in the development of CRC due to genetic and epigenetic changes (Table 1). Three molecular pathways that indicate progression from cancer have been identified: microsatellite instability (associated with hypermutated group), chromosomal instability (completely within the non-hypermutated category), and the CpG Island Methylator Phenotype (CIMP; associated with both the hypermutated and non-hypermutated groups) [14]. These molecular pathways may dictate tumour development and metastatic time, as the epidemiology, mutational trials, and immune responsiveness information vary by route. The methods used to treat an illness might also differ. A biomarker known as “EMAST” refers to elevated microsatellite alterations at selected tetranucleotide repeats recently demonstrated to predict patient survival as a modulator of the three biological pathways. Individuals with sporadic CRC cannot predict whether they develop a metachronous adenoma or cancer based on the previously identified pathways [15,16]. Clinical monitoring is critical in detecting and eliminating metachronous lesions in these patients.

The biological behaviour, prognosis, and therapeutic response of CRC are all very variable. CRC has historically been divided into three subgroups based on the three pathophysiological routes of carcinogenesis: CIN, MSI, and CIMP from a molecular perspective [17]. CIN is the distinguishing feature for most CRCs, accounting for ~80–85% percent of the cases. CIN is characterised by the activation of growth-promoting pathways while simultaneously decreasing the activity of apoptotic pathways, with the latter being more common [18]. These tumours begin as adenomatous polyps due to the APC gene’s deactivation mutation [encodes the WNT pathway effector adenomatous polyposis coli (APC)].

Further, the progress to adenocarcinomas by activating mutations in the KRAS (responsible for the receptor tyrosine kinase signalling) and deactivating mutations in SMAD4 (accounting for the TGFβ, it played an important role in the cell cycle control). Due to the initial event, this pathway is sometimes referred to as the APC pathway [19]. There are no mutations of MSI, MLH1, methylation, or BRAF in the CIN pathway. Moreover, Lynch syndrome (hereditary CRC’s most frequent type) is caused by the germline mutations in the MMR genes, including MLH1, MSH2, MSH6, and PMS2. It is believed that MSI is initiated by a lack in the MMR DNA, causing mutations in the MLH1, MSH2, and MSH6 MMR genes. It is possible to have MMR deficiency due to MLH1 deactivation induced by biallelic hypermethylation of the MLH1 promoter or by dual somatic mutations in the MMR genes [20]. It is noticed that the individual with MMR-deficient and MSI-high CRC (dMMR–MSI-H) demonstrated a good prognosis but did not acquire therapeutics benefits from 5-fluorouracil (5-FU) and has already been characterised based on MSI status [21]. A subset of colorectal cancers known as CIMPs, which are defined by a substantial percentage of CpG island hypermethylation around the promoter of multiple tumour-suppressor genes, is identified by epigenetics rather than by CIN or MSI. Due to its close association with colorectal carcinogenesis’s serrated route, hypermethylation of the MLH1 promoter, as well as its association with female gender, advanced age, and poor histology, CIMP has a significant risk of being found in patients with the disease [22]. However, the lack of a defined definition of CIMP-high makes it difficult to translate this pathophysiological feature into treatment. Although CIMP testing is most commonly done on MLH1, MINT1, CACNA1G, and CDKN2A (could potentially up to 16 distinct genes identified), there is currently does not agree about which cut-off values will be applied to distinguish between CIMP high and CIMP+ on certain scientific procedures must be employed to conduct assessments for CIMP tests [23].

**Figure 2:**
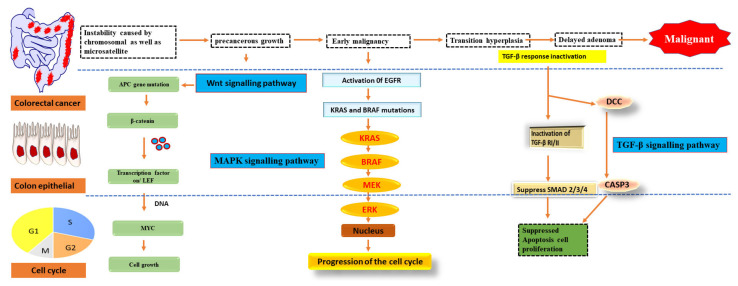
Pathways of signalling that have been genetically changed by CRC.

As early as lesions in the polyp cancer development sequence, gene alterations have a role in cancer creation and progression. The abnormal crypt focus is the initial histological lesion linked to CRC development [24]. Tumour suppressor gene APC is often altered in colorectal cancers, and dysplastic aberrant crypt foci can spread mutations. The Wingless/Wnt pathway is activated by inhibiting APC, a systematic approach to start the polyp cancer development sequence. The Wnt signalling pathway harbours the clonal development of polyp cells to cancer due to subsequent mutations in genes like KRAS or TP53 (Figure 2). The transforming growth factor b (TGFB1)-mediated cell signalling pathway may also be modulated by KRAS and TP53 mutations, further accelerating CRC development [25]. 

Oncogene activation and tumour suppressor gene inactivation are attributed to genetic and epigenetic alterations that occur during colon cancer development. B-Raf sends EGFR signals to KRAS, a proto-oncogene that activates the MAPK pathway [26]. Over half of all CRCs have mutations in KRAS or B-Raf, which trigger the MAPK signalling pathway and promote proliferation while also stopping cell death. The PI3 K, WNT-APC-CTNNB1, and TGFB1-SMAD signalling pathways are also part of CRC. It also includes the RAS–RAF–MAPK pathway [27]. 

The study of epigenetic changes in cancer has led to the identification of genes commonly altered in these specific pathways, leading to in-depth investigations into the biology of CRCs. Novel CRC diagnostic and prognostic tests and new treatments are being developed based on these findings.

## 3. MSI, CpG and CIMP Involvement in the Progression of CRC

**Figure 3:**
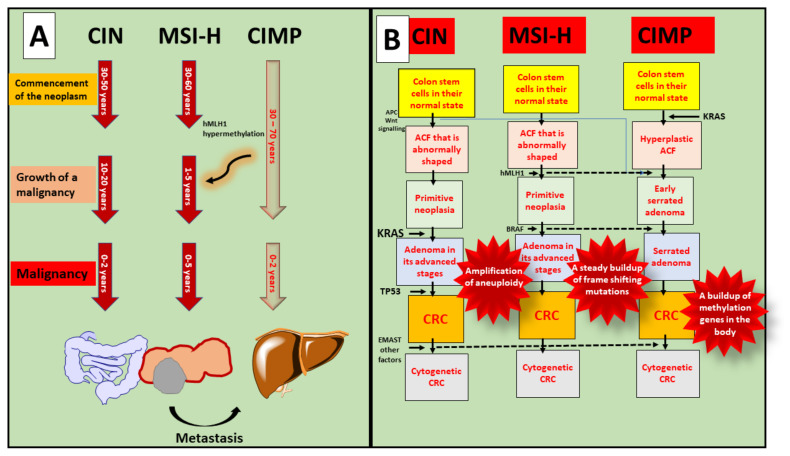
Involvement of MSI, CpG, and CIMP in the progression of CRC. (**A**) Every age groups are susceptible to malignancy, and progression of colorectal cancer shows after the occurrence of metastasis; (**B**) In the case of CIN, various molecular pathways play crucial roles such as Wnt, KRAS, TP53, and EMAST and forms CRC progression. While MSI-H shows progression via hMLH1 and BRAF. Whereas, CIMP is interconnected with KRAS mutation and causes cytogenetic to normal cells.

CRC is a group of several illnesses, some purely molecular, while others include morphological manifestations. Colorectal carcinogenesis has been linked to three distinct molecular pathways, including chromosomal instability (CIN), microsatellite instability (MSI), and chromosomal mismatch repair (CIMP) (Figure 3). For example, chromosomal size and structure are altered in the CIN route. In contrast, changes in the number of mono and dinucleotide repeats in the coding and regulatory regions are seen in the MSI pathway. CRCs grow in two distinct routes, including the conventional adenoma–carcinoma sequence and the serrated neoplasia pathway, morphologically multistep processes [28]. An adenocarcinoma sequence that dates back to antiquity describes the progression of benign adenomas, including tubular adenomas, tubulovillous adenomas, or villous adenomas, progress and transform into invasive adenocarcinomas. The phrase “serrated neoplasia pathway” refers to the concept that sessile or conventional serrated adenomas can develop into invasive adenocarcinomas [29].

Even though Lynch syndrome is associated with inherited MSI-H tumours, the CIN pathway is the underlying biological mechanism for the typical adenoma–carcinoma sequence. Tubular adenomas with variable villosity are Lynch syndrome premalignant lesions. The great majority of spontaneous MSI-H CRCs, in contrast to Lynch syndrome tumours, are considered to develop in CIMP-H sessile serrated adenomas with BRAF mutation through methylation-associated inactivation MLH1. Hereditary and spontaneous MSI-H CRCs take the conventional adenoma–carcinoma sequence and the serrated neoplasia pathway, respectively, whereas MSI-H tumours follow a distinct morphological multistep approach. CIMP-H/non-MSI-H CRCs, in contrast to CIMP-H/MSI-H CRCs, originate from pre-existing conventional serrated adenomas [30].

On the whole, 65–75 percent of CRCs are caused by the CIN route, whereas 20–30 percent are caused by the CIMP pathway and 5 percent by the hereditary MSI pathway. Females have a larger percentage of the CIMP route than men. Older persons have a higher percentage of the CIMP pathway than younger people. The CIMP pathway is more prevalent in the proximal intestine than the distal colon or the rectum [31].

## 4. Epidemiology

**Figure 4:**
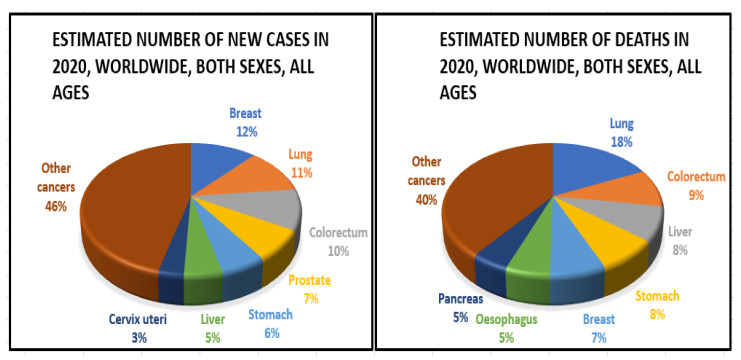
Epidemiology of colorectal cancer.

The frequency of colorectal cancer (CRC) has risen alarmingly in recent years around the globe (Figure 4). In 2020, an estimated 1.93 million new CRC cases were identified, and 0.94 million people died as a result of CRC-related causes globally, accounting for 10% of global cancer incidence (total 19.29 million new cases) and 9.4% of deaths due to cancer (total 9.96 million deaths). It is projected that CRC will be the third-greatest cause of cancer-related deaths in men and women globally in 2020, with 515,637 deaths in men and 419,536 in women, respectively [32]. Over 5.25 million individuals globally (5-year prevalence) have CRC, making it the second-most common disease after breast cancer (7.79 million new cases per year). Approximately 1.15 million new instances of colon cancer, 0.7 million cases of rectal cancer, and 50,000 cases of anal cancer in 2020 were found, according to the database from GLOBOCAN 2020. These figures are expected to rise to 1.92 million, 1.16 million, and 78,000 by 2040. Countries have different rates of colorectal cancer (CRC) [33]. Among countries, the greatest age-standardised incidence rates were observed in Hungary (45.3 cases per 100,000 people in 2020), Slovakia (43.9%), Norway (41.9 cases per 100,000), the Netherlands (41.0 cases per 100,000), and Denmark (40.9 cases per 100,000). Guinea, Gambia, Bangladesh, Bhutan, and Burkina Faso will have the lowest age-standardised incidence rates in 2020 at 3.3, 3.7, 3.8, 3.8, and 3.8 cases per 100,000 people, respectively.

## 5. Biomarkers Based on Epigenetic Changes for CRC

When CRC progresses from an adenomas or serrated lesions stage, genetic and epigenetic changes accumulate in the lesions, giving progressively dysplastic characteristics until they become an adenocarcinoma [34]. To avoid this illness, it is critical to find precancerous lesions and early-onset colorectal cancer in people at average risk who are otherwise healthy during routine screening. As far as CRC screening is concerned, colonoscopy is the gold standard, since it can detect and eradicate precursor lesions [35]. For these reasons, many people choose not to get colonoscopies. They are painful, costly with low compliance rates, and can cause complications, including haemorrhage and perforation. For precursor lesions like adenomas, non-invasive screening procedures like the faecal occult blood test (FOBT) and faecal immunochemical test (FIT), widely used in Europe and other Western nations, have lower sensitivity and specificity than colonoscopy. As a result of these drawbacks, new non-invasive detection methods for precursor lesions and early-stage CRCs are urgently required [36]. 

At intermediate stages of CRC staging, the currently used tumour–node–metastasis (TNM) classification approach is inadequate for prognostication and clinical decision-making [37]. Individuals with a high risk of illness recurrence or mortality (prognostic biomarkers) and those who would benefit from chemotherapy, immunotherapy, or targeted therapy are urgently in need of biomarkers that aid identify these patients (which may be unnecessary). Many epigenetic biomarkers, including DNA methylation, histone alterations, miRNAs, and long noncoding RNA, have shown promise as clinically significant biomarkers for CRC diagnosis, prognosis, and therapy response prediction (lncRNA) [38]. 

**Figure 5:**
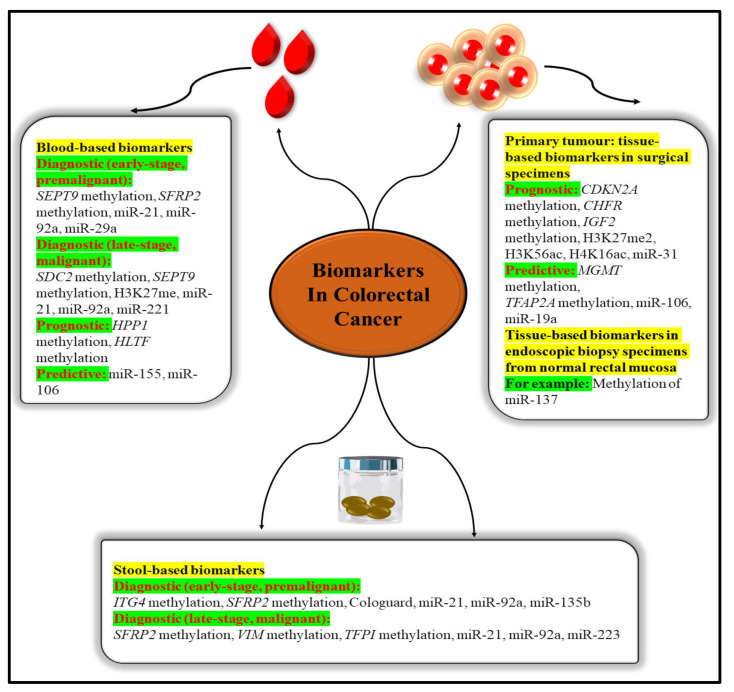
Epigenetic biomarkers in CRC.

Blood and other body fluids and tissues can include quantifiable indicators of pathological or physiological processes or conditions or diseases, such as molecular markers (biomarkers). Molecular markers can be used to identify conditions or diseases. Biomarkers are important diagnostic, prognostic, and therapeutic tools for cancer detection, diagnosis, and therapy selection (Figure 5). It can also be used to pinpoint the exact location of a tumour and identify any areas that are at an advanced stage of growth or are receptive to a particular therapy [39]. These molecular markers represent the mechanisms of neoplastic cell exfoliation and mucus secretion of aberrant glycoproteins in CRC by reflecting the mechanisms of cell exfoliation and mucus secretion. Shortly, researchers hope to find novel, non-invasive DNA, RNA, or protein-based molecular markers that may be used to detect colorectal cancer in the blood, faeces, and other body fluid. The prognostic, predictive, and diagnostic markers have been developed for the determination of the CRC. Diagnostic biomarkers indicate the likelihood of the illness progressing. Patient treatment measures can be predicted with the use of these molecular markers. Predictive biomarkers are utilised for forecasting the efficacy of treatment in cancer patients [40]. Colorectal polyps can be detected early with the use of the following biomarkers.

### 5.1. Molecular Markers for Diagnosis

#### 5.1.1. HNPCC (Hereditary Nonpolyposis CRC)

HNPCC is the most well-known autosomal dominant hereditary colon cancer condition. It is a screening test for the early identification of colorectal cancer (CRC). HNPCC is present when a germline mutation is identified in one of the four MMR genes, such as MSH6, MLH1, MSH2, and PMS2, all found in the human body. Many young patients with a new genetic condition come from families where colon cancer runs in the blood. 

#### 5.1.2. Telomerase

The telomerase is key enzyme that preserves the telomere (repeating DNA (TTAGGG/AATCCC in the human telomeres) that protects from chromosomal breakdown, elimination of operational genes, and cell death. Telomerase is necessary for the maintenance of the telomere. The activation of telomerase is an independent diagnostic and prognostic marker for malignant tumours that can be used independently.

#### 5.1.3. Insulin Like Growth Factor Binding Protein 2 (IGFBP2)

According to research, compared to healthy individuals, CRC patients had higher serum and plasma levels of IGFBP2. It has emerged as a diagnostic device for the primary identification and development of CRC, since it is implicated in cancer cell proliferation, migration, and invasion.

#### 5.1.4. Pyruvate Kinase M2 (PKM2)

PKM2 refers to a cytosolic enzyme expressed in both normal and malignant cells and plays an important function in energy metabolism. PKM2 expression is upregulated in CRC and other gastrointestinal malignancies. It may also be relevant for CRC inquiry and/or diagnosis based on stool or blood samples as a dismal prognostic sign.

### 5.2. Molecular Markers for Prognostic

#### 5.2.1. p53

The existence of the tumour inhibitor p53 is also considered an independent prognostic factor for colon cancer. The alteration of the tumour-suppressor gene p53 is an initial and critical step in ulcerative colitis-associated carcinogenesis (sometimes referred to as TP53). The development of carcinoma in an adenoma coincides with a p53 mutation and a loss of heterozygosity (LOH) in the wild-type allele, further demonstrating its function in the regulation of malignancy. Patients with CRC who are missing this gene have a bad prognosis.

#### 5.2.2. 18 q Loss of Heterozygosity

Patients with colorectal cancer (CRC) who have chromosomal 18q LOH have a reduced chance of survival. Patients with stage II or stage III colon cancer who lose their somatic heterozygosity have a worse prognosis than those whose tumours retain both of their parents’ alleles on chromosome 18 q. Patients with stage II or stage III colon cancer with this 18qLOH have a poorer prognosis than those with normal alleles on chromosome 18q.

#### 5.2.3. MLH1 Methylation

DNA microsatellite unstable recognition or the absence of the MLH1 protein production on immunohistochemical investigation confirm somatic MLH1 inactivation in primary colorectal malignancies, which is more common in early-stage colorectal cancers than in late illness. A more indolent illness or a better prognosis might result from this inactivation in the absence of adjuvant treatment.

#### 5.2.4. VEGF

One of the angiogenic agents in CRC is vascular endothelial growth factor (VEGF), which is expressed in roughly 50% of CRCs but only in extremely low amounts in normal colonic mucosa and adenomas. As a result, VEGF-1 expression appears to deliver useful predictive information in CRC patients. 

### 5.3. Predictive Molecular Markers

#### 5.3.1. KRAS

The discovery of KRAS mutations is employed as the foremost potential prognostic marker in anti-EGFR (epidermal growth factor receptor) antibody-based therapy in CRC, such as panitumumab and cetuximab.

#### 5.3.2. B-Raf V600E

The activation of this kinase by somatic mutation leads to an unconstrained MAPK signalling pathway in humans. The BRAF V600E activating mutation does not respond to EGFR inhibitor treatment in individuals with stage IV CRC. Although the significance of BRAF mutant level as a predicting marker gene is uncertain, BRAF mutant as a biomarker of anti-EGFR antibody resistance is perhaps the extensively researched predicting potential of BRAF alteration.

#### 5.3.3. PIK3CA Status

The PIK3CA mutation causes an abnormal stimulation of the AKT pathway, leading to cancer cell growth. If the PI3K/AKT pathway is persistently activated, it is likely to affect CRC progression significantly. Colon cancer cells often with an induced PIK3CA mutant were more sensitive to cetuximab in preclinical studies even than PIK3CA wild-type cells.

#### 5.3.4. ERCC—1

Repair protein for DNA excision ERCC-1 is a nucleotide excision repair gene that is involved in DNA repair. A new analysis of metastasis and stage II or III CRC and evaluating ERCC-1 as a possible predictive biomarker in direct response to oxaliplatin by studying dysregulation of ERCC-1 expression in between oxaliplatin treatment has been presented [41,42,43,44].

## 6. Diagnostic/Materialistic Tool for the Treatment of Colorectal Cancer

A diet rich in vegetables, fruit, and whole-grain cereal goods, as well as regular exercise, lowers the chance of developing colorectal cancer. CRC risk has been linked to environmental variables such as obesity, smoking, and high alcohol intake. Calcium and vitamin D3 supplementation may have a disease-fighting effect. It has also been shown that long-term usage of acetylsalicylic acid (NSAIDS) decreases the risk of cancer. Colorectal cancer is just one of many cancers that can be prevented by quitting smoking or abstaining from smoking. Adenomas (primary prevention) and malignancies (early detection) can be detected with screening tests (secondary prevention).

**Figure 6:**
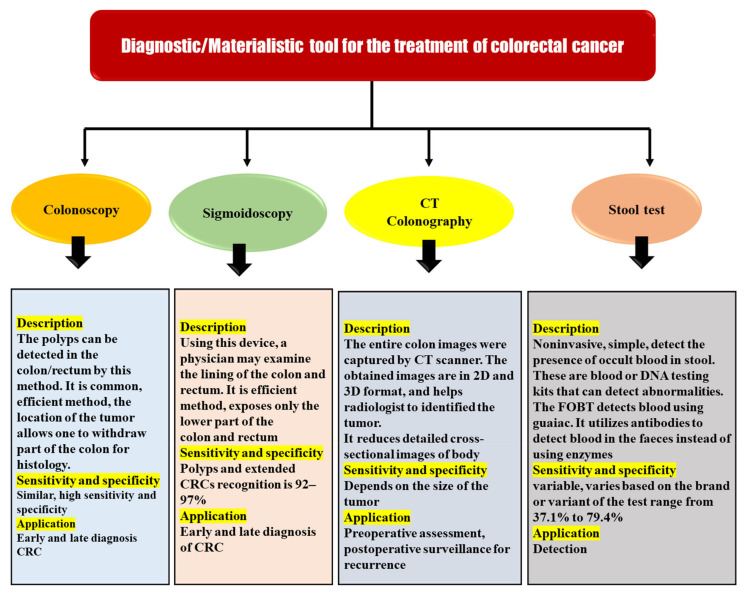
Diagnostic/materialistic tool for the treatment of colorectal cancer.

This is why regular screenings in high-risk people can help detect CRC even if symptoms are not present. After the diagnosis of colon cancer, the diagnostic tools are also useful for the TNM staging, which is used to determine the appropriate treatment. These include colonoscopy, sigmoidoscopy, magnetic resonance imaging (MRI), CTCG, trans-rectal ultrasonography, stool testing, double-contrast barium enema, and many others. However, all techniques have some of the other related limitations and remain inaccurate for effective diagnosis in the early phases of the disease process. There is a pressing need to develop more advanced diagnostic methods in order to increase survival rates, reduce disease-related problems due to diagnosis at later stages, and properly stage the disease so that appropriate therapy may be prescribed. Here are some of the most commonly used diagnostic tools, along with their advantages and disadvantages (Figure 6) [45,46].

### 6.1. Colonoscopy and Sigmoidoscopy

Endoscopy is the most often used and most effective procedure for CRC diagnosis. Colonoscopy and sigmoidoscopy are included in this procedure. These tests enable the detection of a tumour and the removal of a portion of the large intestine for histological analysis to be performed. Sigmoidoscopy has a sensitivity and specificity of 92–97 percent for the detection of polyps and expanded CRCs. Sigmoidoscopy only reveals the lowest portion of the colon and the rectum when performed with a flexible endoscope. Colonoscopy provides the same level of sensitivity and specificity as a colonoscopy in terms of obtaining an image of the entire intestine. Colonoscopy has several advantages over other screening tests, including the fact that it may be completed in a shorter period and that it improves the patient’s acceptance and tolerance of new sedative procedures. In comparison to the control group, the study that included individuals with an average risk of CRC following colonoscopy revealed a 67 percent of overall decrease in morbidity and a 65 percent of the overall decrease in mortality. Preliminary screening with sigmoidoscopy and colonoscopy was found in a randomised trial to reduce CRC incidence and mortality by 33% and 38–59%. As a result of this discovery, sigmoidoscopy has become increasingly popular as the main screening method in England and elsewhere. Smaller polyps (6–10 mm) are difficult to identify with this invasive method. It also restricts cancer detection in the ascending colon, the transverse colon, and the cecum. Endoscopy (sigmoidoscopy) can be used to diagnose and treat CRC in patients who are ineligible for surgery (according to the severity of the tumour or comorbidities). Methods for removing the cancer-related blockage are among these procedures. Patients typically report discomfort from endoscopy despite its many benefits. Due to the potential for consequences, people avoid or postpone the evaluation. Virtual colonoscopy has been increasingly popular in recent years. The use of computed tomography is it provides a 3D view of the large intestine. Perforation or bleeding from the large intestine can be reduced by the use of virtual colonoscopy [45,47,48].

### 6.2. CT Colonography

When referring to a computerised x-ray imaging procedure, “virtual colonoscopy” or “CT colonography”, both terms refer to the same thing: a method that produces two- and three-dimensional images of the colon. It is possible that CT colonography can be used as an alternative to colonoscopy in patients with obstructive or stenotic tumours. After the colon has been completely evacuated (bowel preparation), it is insufflated with air or carbon dioxide to increase its size for better imaging during the CT scanning procedure. After insufflation, the subject is scanned in both the supine and prone positions. CT colonography is comparable to colon endoscopy in terms of patient pain because of the need for colon evacuation and insufflation, but it has the advantage of not requiring anaesthesia. For polyps larger than 10 mm in diameter (big polyps), the detection sensitivity and specificity are excellent (82 percent), but the sensitivity is poor (63 percent) for polyps between 6 and 9 mm in diameter (small polyps). Furthermore, the method cannot distinguish between flat and serrated lesions. For diagnosing the local stage of a CRC tumour, MRI is preferred over CT colonography, which performs poorly in this regard. Due to its high level of accuracy in determining the tumour’s invasion and spread, preoperative MRI is useful in planning surgery. The CTCG approach has many drawbacks, including radiation exposure, a lack of sensitivity to detect small polyps, the necessity for intestinal preparation, and the need for an extra procedure to remove polyps if they are discovered. As a result, the patient’s concern rises and the expense rises as a result of unneeded monitoring, which is exacerbated by extracolonic lesion identification [45,47]. 

### 6.3. Stool Test

GFOBT and FIT are two of the most commonly utilised main screening modalities for CRC detection. Stool testing can identify blood spilling from CRCs in the stools of asymptomatic patients. FITs are more commonly used than gFOBTs because of their ease of use and greater sensitivity. Patients who have a positive stool test must have a colonoscopy to get an accurate diagnosis and to remove any polyps that are found. The detection of DNA markers released by malignant cells in a person’s stool is a promising technique for CRC screening. Stool DNA testing has a high level of sensitivity (71–91%) and specificity (93–100%) for the identification of colon cancer. False results are common because of the low sensitivity and specificity of the FOBT. Additionally, these tests fail to detect nonbleeding tumours. Faecal occult blood testing is a straightforward, inexpensive, and non-invasive diagnostic procedure. Haemoglobin in faeces implies gastrointestinal bleeding, according to the test. Since it can come from both malignant alterations and polyps larger than 1 to 2 cm, blood in faeces is an unspecific sign of colon cancer. The test’s sensitivity can be increased by as much as 90% by repeating it. Heme and human globin are the two building blocks of haemoglobin, and the immunohistochemistry faecal occult blood test (FIT) confirms its presence in CRC diagnoses. Aside from sDNA, which indicates DNA changes in colorectal adenocarcinomas, molecular diagnostics, such as PCR, may be used to separate and discriminate DNA from that of bacteria in faeces. The usefulness of CRC molecular diagnostics based on genetic and epigenetic testing is restricted. Costs are expensive, because it is not widely available. Molecular diagnoses of CRC have some drawbacks, which have prompted researchers to look for biomarkers that can be found in biological samples at a cheaper cost [45,47,49,50].

## 7. Nanotechnology-Based CRC Diagnostic Techniques 

Medicines based on nanoparticles are being developed as therapeutic methods for cancer therapy, which has led to significant improvements in pharmaceutical research [51]. These advancements have reduced the adverse effects of cytotoxic drugs while simultaneously increasing their efficacy and solubility. The encapsulation of anticancer cargo, including as siRNA, antibiotics, and chemotherapeutics has proven highly effective in the last 50 years using a variety of distinct nanoparticles with various forms, sizes, and chemical natures [52]. Using the increased permeability and retention effect afforded by tumour vascular and lymphatic drainage, these first-generation anticancer nanoparticles passively penetrate tumour tissue, allowing nanoparticle extravasation and storage within cancer cells, improving therapeutic effectiveness. Early diagnosis and therapeutic efficacy monitoring can both benefit from the use of nanoparticles [53]. Incorporating various contrast agents (such as radioactivity, superparamagnetic, or fluorescent), as well as targeting groups and biocompatible coatings, into a nanoparticle design is a feasible option [54]. Due to the disadvantages of low tissue specificity, rapid clearance, and nonspecific extracellular distribution associated with small molecular weight gadolinium and metal chelate-based contrast agents, nanotechnology may be used to modify these contrast agents to improve CRC diagnostic sensitivity and specificity [55]. Various nanostructures are being used as inputs of in vitro diagnostic assays for protein indicators or nucleic acid targets. In vivo imaging with nanostructures, similar to in vitro diagnostics, seems to be a potentially intriguing area in diagnosing colon cancer [56]. There are, however, just a few examples of how nanotechnology has been used in CRC imaging. Novel MRI contrast agents are among the most readily useful pieces of colorectal cancer technology. The introduction of nanostructures that modify standard contrast agents like gadolinium or imaging agents like iron oxide could increase the detection capability of medical image [57]. These nanostructures enhance the capabilities of traditional MRI imaging, but they also provide new possibilities for the early diagnosis and healing of the CRC. Tumour early diagnosis and healing techniques not possible with current conventional technologies have been found as beneficial nanotechnological applications in cancer biology [58]. When it comes to diagnosing, producing, and healing some malignancies, nanometre-sized particles of various structures and conformations have shown to be highly effective and promising new approaches. A patient’s long-term survival can be affected by the early identification of CRC, which is critical for prevention [59]. Here are a few examples of recent Nps accomplishments that have opened up new possibilities for the early identification and successful treatment of CRC.

### 7.1. Quantum Dots (QDs)

QDs are semiconductor nanocrystals that generate fluorescence when excited by light and have remarkable optical properties, such as high brightness, photobleach resistance, and the ability to emit fluorescence at many wavelengths [60]. QD-based nanotechnology, with its optical and chemical benefits, is a developing platform for cancer research, particularly colorectal cancer [61]. Its bandgap energy, the degree of energy required to transport electrons from one electronic zone to the next, this time at a greater frequency, is an important characteristic of a semiconductor nanocrystal. An exciton is a pair of electrons and holes formed as a result of this excitation strategy. The unstable exciton returns its ground state and releases fluorescence photons in the form of energy [62]. When the semiconducting nanocrystal size and shape approach the bulk Bohr exciton radius, which is still generally 2–10 nm in length, electrical and optical properties are added to the particle. Scientists know that as a nanocrystal’s size decreases, its bandgap increases, and its stimulation and emissions wavelength decrease because of this inverse relationship [63]. 

Park et al. used an enzyme-sensitive fluorescent dye in conjunction with antibody-QD conjugates to produce a multiplexed detection method for the fast and precise diagnosis of CRC. The instrument incorporated a Cresyl violet–glutamic acid (CV–Glu) derivative as the fluorescent molecular probe, as it easily transits between two fluorescent colours in reply to k-gluta myltranspeptidase enzymatic activity (GGT). A biocompatible, 690-nm emitting AgInS_2_/ZnS (core/shell) QD probe was created by bioconjugating an anti-matrix metalloproteinase-14 antibody (MMP14). The probe provided significant findings in ex vivo and in vitro applications by employing human CRC cell lines, ex vivo patient tumour samples, and ex vivo experimental animal model tissues. The researchers utilised two-photon microscopy to show that the co-application of both probes penetrated the tissue at depths of 10–20 mm quickly. The study’s findings indicated that using both probes simultaneously allowed for quick (within 5 min) and accurate imaging of tumour lesions that would otherwise be impossible to identify using traditional colonoscopy procedures. When CRC was in its beginning phases, the dual-probe approach helped find hyperplastic polyps and adenomas [64].

Carbary-Ganz et al., created a dual-modality imaging technique that uses optical coherence tomography and laser-induced fluorescence to provide non-destructive endoscopic viewing of CRC with just a minimal amount of invasiveness. While CRC develops and progresses, this method allows for the concurrent longitudinal surveillance of biochemical and morphological changes. With great sensitivity and specificity, QDot655-VEGFR2 localised to the colon in carcinogen-treated animals and provided a significant difference between sick and healthy tissue ex vivo [65].

### 7.2. Iron Oxide Nanocrystals

Iron oxide NPs are incorporated in MRI as a contrast agent for human use because of their dual magnetic and photothermal properties. Apart from that, they are highly biodegradable in vivo, and then, the iron salts delivered after disintegration can be ingested into the body system through a well-coordinated biochemical mechanism [66]. There is a magnetic core to the iron oxide nanocrystals and a polymer covering, which will contain the various medicinal molecules. These nanoparticles have various biological properties due to their relatively small dimension, efficient surface region, minimal deposition rate, and ease of cellular transport [67]. The severe toxicity and oxidation sensitivity of strong magnetic metals like cobalt and nickel seem to offer limited biomedical utility. Using nanoparticles, a cancer cell is transformed into a tiny magnetic magnet that may be attached to the biopsy needle’s tip. These nanoparticles are unstable in aqueous environments and easily collected and deposited [68]. It cannot detect the target tumour using nanoparticles of the iron oxide in vivo due to the diverse overexpression of the highly targeted receptor in cancer cells. Using magnetic nanoparticles, it is possible to discriminate between malignant and normal tissue. It is possible to create functional groups on these nanoparticles that crosslink with tumour-targeting molecules such as peptides or monoclonal antibodies or small molecules for the diagnostic testing or remedial agent administration due to their great surface area [69].

Kim et al. used a 3D multi-echo gradient echo to compare the particular performance of three distinct parametric techniques (normalised signal intensity, R2* (R-lymph node Region of interest), and susceptibility) to measure the number of ultra-small superparamagnetic iron oxide (USPIO) particles in lymph nodes. Before and after USPIO injection, nine rabbits with VX2 tumour implants were scanned. All of this was accomplished by combining 3D GRE amplitude and phase data to produce multi-echo combined T2* (weighted multi-echo combined magnitude image of 3D mGRE 24 h after USPIO injection)-weighted pictures and an R2* map. USPIO build-up in 18 lymph nodes (nine metastatic and nine reactive) was found to have a significant impact on signal intensity. R2* differences before and after USPIO injection were statistically significant between lymph nodes that were reactive and metastatic (*p* < 0.05), although signal intensity and vulnerability were not significantly different between the nodes. USPIO-enhanced MRI employing R2* mapping from 3D multi-echo GRE may be used to identify lymph node metastases and to perform a parametric analysis of the lymph node status in a rabbit model, as demonstrated in their work [70]. 

Wu et al. conducted a meta-analysis to examine how well USPIO-enhanced MRI and non-enhanced MRI, as well as USPIO-enhanced MRI in various body areas and postcontrast MRI performed in the diagnosis of lymph node metastases. As a result of this meta-analysis, USPIO-enhanced MRI is more accurate than conventional MRI in the diagnosis of lymph node metastases. For lymph node characterisation, postcontrast pictures alone are sufficient to provide the same diagnostic performance as pre-contrast MRI. There is still a lot of research to be done on the use of USPIO enhanced MRI in clinical practice [71].

Kuo et al. developed an antibody-targeting peptide AP-1(MPVA-AP1) and anticancer medicines by incorporating the magnetic nano-vehicles. The magnetic nano-vehicles disseminated in the dilute solution demonstrated great hemocompatibility and toxic-free to L929 fibroblasts, indicating that they might be used in treatments. The researchers found significant encapsulated hydrophobic and hydrophilic low-molecular-weight medicines and protein-like pharmaceuticals using a straightforward manufacturing approach. Magnetic nano-vehicles were also used to immobilise the antibody-targeting peptide AP-1, which was verified using electron spectroscopy. The results of a CRC cell (CT26-IL4Rα) assay demonstrated that the AP-1-bound nano-vehicles (MPVA-AP1) show exceptional targeting and selectivity. In the absence of a magnetic stimulation, a stable storage test revealed virtually little leakage of the encapsulated medicines. Instead of rupturing in the presence of a high-frequency magnetic field, doxorubicin-loaded nano-vehicles released the drug with pinpoint accuracy. There was further evidence from in vivo experiments that the magnetic nano-vehicles showed significant chemotherapeutic and thermotherapeutic effects. Due to this, magnetic nano-vehicles with smart properties, such as MPVA-AP1, hold great promise for anticancer applications that need precise dosing and regulated release [72].

It was found that the coprecipitation technique proposed by Syu et al., (2019) was simple and ligand-assisted to manufacture biocompatible iron oxide (IO) nanocrystals with NIR absorbance for cancer theranostics. Nanocrystals had little cytotoxicity in HT-29 colorectal cancer cells; however, the vitality of cells treated with nanocrystals dropped substantially following laser irradiation at a 808-nm wavelength. Cell death is thought to be caused by changes in protein secondary structure and membrane permeability, among other things. Nanocrystals with magnetic field (MF) application significantly enhanced tumour aggregation by fourfold in the in vivo studies, leading to a thrice-larger T2-weighted MR signal than that provided by a conventional T2-weighted MR contrast agent (Resovist^®^) and improved photothermal potency for the therapy of cancer. The novel iron oxide nanocrystals were shown to be highly biocompatible and show significant promise as a cancer theranostic agent [73].

## 8. Synthetic/Herbal Nanocrystal Used in Drug Delivery System

Biomedicine’s use of nanotechnology is expanding quickly. The field of nanotechnology serves as a link between various NPs and nanophases in terms of both technology and science [74]. Particles with a size range of 1–100 nm with great specific surface area and specialised surface properties are referred to as nanoparticles (NPs) [75]. It is possible to obtain controlled drug release, site-specific drug delivery, and reduced toxicity by using nanoparticles (NPs). The NP’s size significantly impacts how well it is taken in by cells and excreted from them in vivo. It has been demonstrated that greater particulates have much reduced concentrations of intracellular delivery than small nanoparticles at a similar quantity and that NPs with such a dimension of 100 nm had 2.5-fold more absorption than 1-m size nanoparticles [76]. Since the glomerular capillary sieve coefficient is 10 nm, NPs with a smaller diameter exhibit better renal clearance. Larger NPs, on the other hand, are unable to diffuse into intracellular gaps because of the increased permeability and retention (EPR) effect, lacking in a approach cancer cells [77]. The toxicology of NPs is influenced by physicochemical characteristics, including size, shape, and concentration, which affect absorption, circulation time, and total toxicity. The phospholipid bilayer structure gives the cell membrane a negative surface charge, making it even more susceptible to positively charged particles [78].

In other words, because they immediately enter the cell membrane and cause membrane rupture or damage, highly positive charged NPs have higher cytotoxicity. NP surfaces have been modified to hold a negatively charged initially to allow them to the exist specific macrophage organelles that is later converted to a positive charge by environmental cues to enhance drug administration [79]. To counteract the cationic charge, scientists used serum albumin in preincubated NPs, including shape and size, toxic effects, quantity, and charge density, which should be considered while designing nanomedicine. For example, MRI imaging, cancer diagnostics, precise cell and subcellular treatment, and the detection of genetic mutations NPs have enormous promise, with suitable surface changes in biomedical and therapeutic applications such as nanoparticle surface modification [80].

## 9. Phytomedicine Drug Delivery Using Nanoparticles

Developing resistance to chemotherapeutic drugs and radiation therapy is a key challenge in cancer treatment. Phytochemicals with low side effects, namely lycopene, genistein, epigallocatechin gallate, anthocyanin, curcumin, and resveratrol, are being investigated by researchers as an alternative therapy to help overcome this problem [81,82]. On the other hand, phytochemicals have several disadvantages, including poor absorption, limited water solubility, and rapid metabolism. Consequently, using NPs to formulate these phytochemicals can improve phytochemical medication delivery while also minimising adverse effects [82].

### 9.1. Curcumin

As a chemoprevention and anticancer agent, curcumin obtained from Indian spices turmeric is highly effective. Inhibiting different signalling pathways such as NF-κB, STAT3 and EGFR, which contribute to tumour development and metastasis, slows the advancement of many malignancies, including CRC [83]. Due to the nanoformulation, curcumin has improved water solubility and distribution while causing no harm to healthy cells. To create a nanocurcumin formulation for use in CRC treatment, NPs made of liposomes, polymers, cyclodextrin, nanogels, gold, lipids, and micelles can be used [84].

In addition to its ability to act as a free radical scavenger, curcumin also changes the expression of several stress proteins and angiogenesis-related genes. It inhibits the activity of numerous key transcription factors, namely activator protein 1 (AP-1) and nuclear factor κ-light chain enhancer of activated B cells (NF-κB) [85]. Additionally, the size of 10 μm showed significant antioxidant activity. It triggered cell death when they reached a concentration of around 50% at 50 μm, which may be due to the production of superoxide radicals [86]. Human plasma levels can be as low as 0.41–1.75 μmol/L after taking 4–8 g of turmeric orally each day. The curcumin concentration is critical for evaluating the biological consequences of frequent oral intake. Bio-transformed moieties, such as tetra hexahydro–curcumin, have drawn attention because of their poor systemic bioavailability [87].

**Figure 7:**
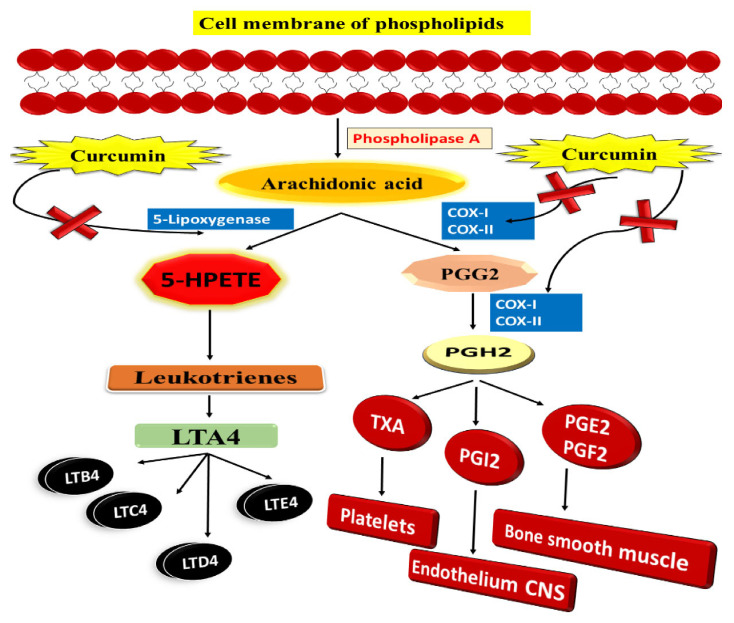
Curcumin and the arachidonic acid metabolism: A potential inhibitory effect.

Additionally, research shows that curcumin is an efficient chemopreventive drug in addition to its antitumour properties (Figure 7). It works by inhibiting cyclooxygenase, phospholipase A2, and phospholipase-Cr1 in tumours of the colon, stomach, and skin. It is clear that antagonists of COX-2 are effective in suppressing polyp development and cancer growth in FAP, and were it not for the negative consequences, COX-2 inhibitors would be strongly suggested for cancer polyp chemoprevention [88]. In order to avoid carcinogenesis, we require drugs that block cellular pathways that cause or promote cancer. With these characteristics, curcumin is a promising chemopreventive agent. It has recently been shown that 5-LOX plays a crucial function in controlling cell proliferation and apoptosis [89]. Some research shows 5-LOX overexpression in cancer cells and human tumours, such as those of the gastrointestinal tract and liver and those of the breast and prostate. 5-LOX has also been shown to be overexpressed in the brain and the mesothelium. 5-LOX overexpression has been connected to enhanced proliferation and tumour growth in investigations done in the lab and on mice in the wild [90]. 

Ohd et al. indicate that 5-LOX and cysteinyl leukotriene receptors are overexpressed in human colon adenocarcinomas and that individuals with these mutations have a poor prognosis. It indicates that increased 5-LOX expression is related to malignant transformation, although the exact processes that link 5-LOX gene expression to colon cancer development are unclear. The use of COX-2 knockout mouse models has demonstrated that COX-2 plays a function in colon carcinogenesis. However, in colon carcinogenesis, similar data from 5-LOX knockout animals are missing [91]. According to Raveendran et al., curcumin-encapsulated PCL exhibits hydrophobicity and effective NP stability due to its amphiphilic block copolymer nature. It was an effective technique, because it targeted the tumour and made tumour cells more susceptible to treatment resistance. Additionally, this formulation enhanced water solubility, resulting in increased in vitro curcumin absorption by Caco-2 CRC cells. There was significantly less inhibition in the micelles of bigger amphiphilic block copolymers than smaller ones [92].

### 9.2. Resveratrol

**Figure 8:**
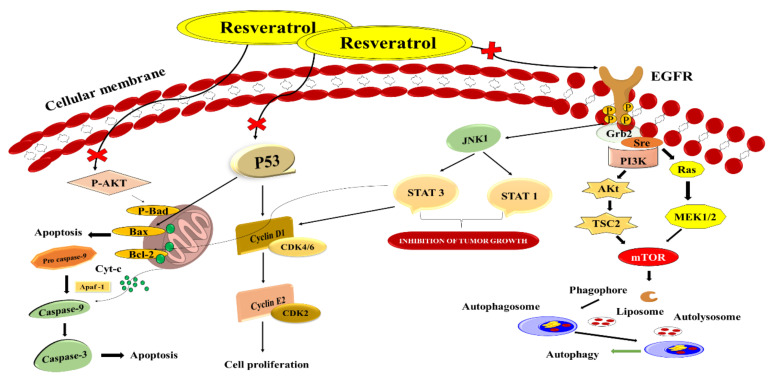
Schematic illustration of resveratrol as a novel healing method for treating CRC by targeting distinct signalling pathways.

Natural phenols such as resveratrol are found in fruits such as red grapes, blueberries, and raspberries [93]. Resveratrol has been discovered to have anticancer, anti-cardiovascular, anti-inflammatory, and antiaging biological and pharmacological effects [94]. Further studies have shown that resveratrol has antiproliferative properties in vitro against CRC via modulating intracellular signalling pathways such as PTEN/PI3K/Akt, Wnt/β-catenin, and others [95]. It is possible to discover therapeutic medication’s biological targets and molecular processes by using network pharmacology as an approach in clinical illnesses (Figure 8). As a result, identifying biomarkers and treatment molecular pathways is crucial even before diagnosing a patient with a relapsed CRC [96]. Resveratrol belongs to the phytoalexin stilbene compound and is isolated from the various plants extracts. Chemo-sensitising, antioxidative, and anti-inflammatory characteristics are some of the things it does. Due to the poor physicochemical profile of resveratrol, it is not generally recommended therapeutically despite its positive features [97]. The iNOS, COX-2, and other inflammatory signalling pathways are all reduced by resveratrol, inhibiting proinflammatory mediators, including IL-1β and TNF-α [98]. Soo et al. created and improved an innovative drug carrier by co-encapsulating resveratrol and cyclodextrin-resveratrol inclusion complexes in liposomes’ lipophilic and hydrophilic compartments. The final formulation’s particle size, polydispersity index, and zeta potential were measured at 131 ± 1.30 nm, 0.089 ± 0.005 and −2.64 ± 0.51 mV, respectively. The drug release profiles of 40–60% were found to be acceptable for the standard liposomal formulations and free resveratrol, while the innovative nanoformulations demonstrated total (100%) release in 24 h. It remained steady at 4 °C for 14 days. In HT-29 colon cancer cell lines, resveratrol-encapsulated liposomes showed in vitro cytotoxicity. Liposomes were shown to have a dose-dependent cytotoxicity profile that was also boosted compared to free resveratrol (in DMSO). Using liposomal formulations to deliver hydrophobic chemotherapy agents is possible because of the study’s findings, which show that the co-encapsulation of resveratrol in its purest form with its cyclodextrin complex is feasible [99]. Summerlin et al. enclosed resveratrol in colloidal MSN, and they discovered that the encapsulation efficiency and loading capacity of MCM-48–resveratrol were superior. In CRC cell lines, the formula promoted tumour cell killing via the PARP and cIAP1 pathways. Furthermore, it slowed down the production of NF-κB. As a result, this new approach to medication development may be useful in the future [100].

## 10. Chemotherapeutic Drug Delivery Using Nanoparticles

Most cancers are still treated with radiotherapy and chemotherapy. It is a serious issue that a combination of high dosages and combinational therapy produces severe toxic effects to healthy colon cells. Moreover, it is also associated with a variety of cardiac and digestive side effects. Hence, it is required to reach the target location because of the short half-life of these therapies and the tendency towards resistance [101]. Since the drug’s circulation time and pharmacokinetic characteristics may be improved via nanocarrier-based delivery, it may overcome these adverse effects [102].

### 10.1. 5-Fluorouracil (Capecitabine)

**Figure 9:**
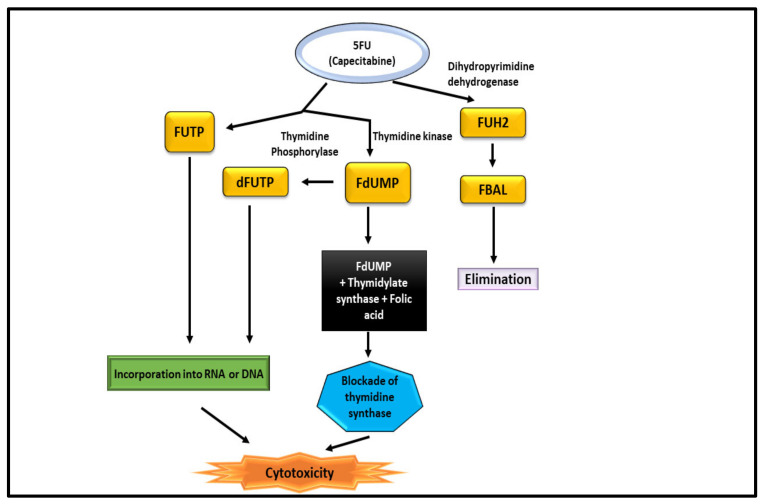
Mechanism of 5-FU for the treatment of CRC.

5-Fluorouracil (5-FU) was applied to heal CRC as a single agent and in combination with other drugs for more than 40 years [103]. Researchers had to develop an oral version of 5-FU because of its short half-life, the necessity of using a central line, and the demand for continual infusions [104]. When compared to conventional 5-FU, Capecitabine provides many benefits. After passing through the digestive tract, it undergoes three enzymatic processes before transforming to 5-FU (Figure 9). Thymidine phosphorylase (TP), the pathway’s last enzyme, is thought to be present in tumour tissue at disproportionately high levels, which increases the agent’s effectiveness and tolerance through tailored delivery [105]. Most CRC chemotherapy regimens start with 5-FU as the main active ingredient. Deoxythymidine monophosphate (dTMP) plays key role in DNA replication and repair and is inhibited by 5-FU by altering into fluorodeoxyuridine monophosphate (FdUMP) in cells [106]. FdUMP creates a stable complex mostly with the enzyme thymidylate synthase, inhibiting the production of dTMP. It often interacts mostly with the formation and activity of nucleic acids by substituting uracil or thymine in DNA and RNA as a secondary method of action. The 5-FU-related gene TYMP (formerly known as ECGF1), genes for the thymidine phosphorylase (TP) protein, varied amongst the groups. After being converted to fluorodeoxyuridine (FUDR) by the action of TP, the active metabolite FdUMP is produced. In vitro, the overexpression of TP increases the 5-FU sensitivity, which is thought to be due to the increased production of FdUMP as a result [107].

Capecitabine was used in a phase II study by Mizumoto et al. The quality of life and disease-free survival of individuals treated with Capecitabine may improve if the adverse effects are reduced throughout the drug. Capecitabine (2500 mg/m^2^/day) was given to CRC patients for five days, followed two days off (5 days on/2 days off regimen). It took three weeks to complete one course, and it took 24 weeks to complete all eight courses. Retrospectively, included patients on the traditional regimen received substantially more treatment courses than those on the 5 days on/2 days off regimen, with a *p*-value of 0.0438. There was a substantially greater rate of patients in the 5 days on/2 days off regimen completing their planned therapy (*p* = 0.0389). However, according to the results of this phase II research, toxic effects related to the 5 days on/2 days off regimen are less severe than those associated with the more traditional regimen, and adverse events are more common. However, there are fewer reports of high-grade toxicities. The novel regimen had a shorter time to treatment failure and showed high practicality. There was no significant difference in overall quality of life or side effects (mainly mild); therefore, the 5 days on/2 days off regimen must be a plan to be studied in future randomised controlled trials to confirm its viability [108].

### 10.2. Oxaliplatin

**Figure 10:**
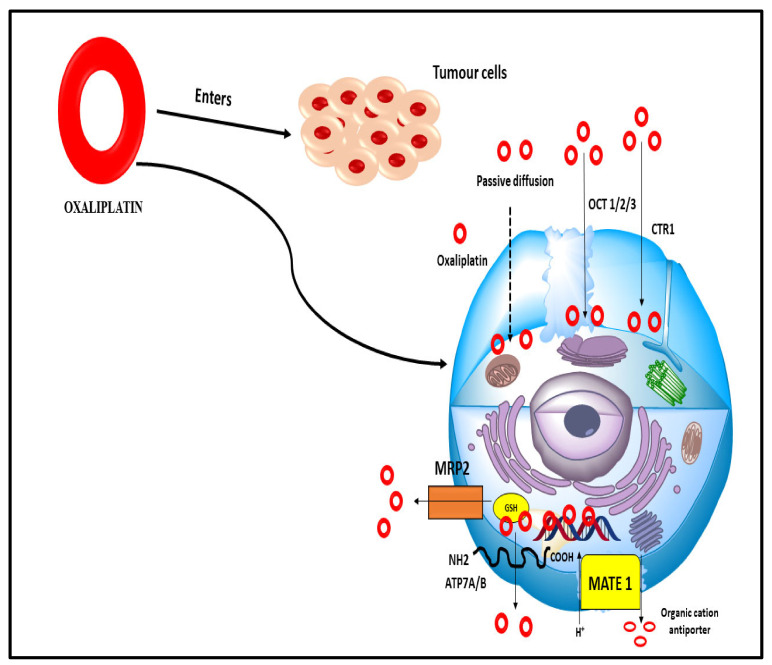
Numerous influx and efflux transporters that allow oxaliplatin to enter the cell passively (dashed arrow). There are several kinds of organic cation transporters, such as the OCT 1/2/3 transporters, copper transporters, MRP2 multidrug resistance-associated proteins, glutathione transporters, MATE 1 multidrug and toxin extrusion transporter, and ATP7A/B ATP-binding cassette transporters.

Oxaliplatin is a third-generation diamino cyclohexane (DACH) platinum compound. Oxaliplatin creates intrastrand connections between two adjacent guanine residues or between guanine and adenine, inhibiting DNA replication and transcription [109]. Oxaliplatin is the third generation of the DACH platinum compound. Oxaliplatin is successful in the treatment of colorectal cancer, but platinum compounds cisplatin and carboplatin have been shown to be ineffective [110]. It is frequently used to treat patients who have not responded to 5-fluorouracil (5FU)-based therapy. Oxaliplatin’s cytotoxic effects, on the other hand, are still poorly studied in detail. Many chemotherapeutic drugs, including oxaliplatin, have been demonstrated to cause apoptosis in tumour cells [111]. Since a medication’s cellular target is dependent on the accumulation in cancer cells, the clinical efficacy of oxaliplatin may be influenced by variables that regulate its accumulation due to its expression by tumour cells or by biological barriers that impact drug disposition. (e.g., enterocytes and hepatocytes) (Figure 10). 

Oxaliplatin can enter cells passively or by transporters that carry it in from the outside. Its physicochemical properties are important to determine if passive diffusion is preferable to other routes [112]. Due to these characteristics, it may accumulate better in tumour cells than cisplatin, even in drug-resistant cells with a lower permeability toward the more hydrophilic drug. Efflux and influx transporters work together to determine how much medication is stored in cells. Oxaliplatin’s therapeutic efficacy and side effects may be influenced by the tissue-specific expression of influx transporters or decreased efflux transporter activity when these variables are present [113]. 

Trifluridine/tipiracil FTD/TPI and oxaliplatin were used synergistically by Limagne et al. to enhance the immunogenic cell death (ICD). Even though all therapy groups indicated T-cell dependence, only the combination effectively induced ICD in vivo. Furthermore, neither FTD/TPI or oxaliplatin affected regulatory T cells or myeloid-derived suppressor cells. However, they did remove type-2 tumour-associated macrophages (TAM2), resulting in increased cytotoxic CD8+ T-cell infiltration and activation. Secondary T-cell exhaustion was related to both tumour cell PD-L1 expression and CD8+ T-cell PD-1 induction.

Last, but not least, even when used alone, anti–PD-1 did not boost the antitumor efficacy of FTD/TPI or oxaliplatin monotherapy, but when combined with the drugs, it did. FTD/TPI and oxaliplatin depleted TAM2 in a new immunomodulatory manner. A rationale for using oxaliplatin and FTD/TPI to remove immunosuppressive cells and boost checkpoint efficacy in patients with metastatic colorectal cancer has been provided by the induction of ICD in vivo by combining these medicines [114].

## 11. Current Colorectal Cancer Clinical Trials and State of Nanotechnology 

Clinical endpoints and biomarkers are two issues that were particularly interested in when it comes to contemporary clinical studies. The alterations in OS amid two treatment groups in research evaluating a single line of therapy are becoming increasingly challenging to accomplish in advanced cancer patients with all types of clinical end goals [115]. There are a rising variety of viable therapeutic choices available in second and subsequent chemotherapy lines in addition to the regularly utilised and often ethically obligatory salvage therapies. However, the initial treatment effect may be weakened or confounded, eventually precluding the proof of a statistical significance overall survival improvement [116]. A growing number of patients will have to be included in clinical studies over an extended period to obtain significant statistical and clinically acceptable OS impacts from long-term patient survival and the ensuing mild OS risk ratio variations within the treated group [117]. Induction chemotherapy combined with a targeted drug is the standard treatment for metastatic colorectal cancer [118]. Clinical trials have evaluated various techniques for maintaining cytotoxic treatment until progression against periods of observation or using various maintenance medicines in several randomised trials. On the other hand, clinical trials have shown wildly divergent effectiveness findings, making it difficult to draw any firm conclusions about which method is best. Table 2 shows the summary of various current and finished clinical studies of CRC.

## 12. Future Prospects and Recommendation

Colorectal cancer is still the leading cause of cancer-related death in the globe. To enhance the overall survival, minimise the disease-free progress, and reduce the recurrence risk, early detection is critical. Oncogenes and tumour suppressor genes undergo successive mutations in the adenoma–carcinoma pathway, progressing from normal to dysplastic epithelium. Normal glandular epithelium becomes adenocarcinoma when genetic and epigenetic alterations to the genome accumulate. Even though the molecular processes responsible for the cancer’s growth and spread remain a mystery, numerous genetic pathways have been postulated to explain CRC aetiology. A biomarker is a useful tool for detecting diseases early on and predicting how well a patient will respond to therapy. Although biomarkers employed at various stages of the illness make it easier to control CRC, each one has its limits. To make advances in biomarker creation and validation, understanding molecular interactions that aid pathogenesis is necessary. The use of nanotechnology in the treatment of colorectal cancer is critical. When it comes to the elimination or, at the very least, the chronic treatment of cancer, nanotechnology has shown to be a vital and optimistic technique. The use of nanotechnology in cancer diagnosis and therapy has been game-changing. It has been a priority in biomedicine to create drug delivery techniques that can affect biodistribution, tissue absorption, and pharmacokinetics, among other things.

Contrary to popular belief, nanomedicine can have a significant impact on human CRC diagnosis and treatment and even improve normal human physiology in some cases. We hope that this article will help scientists, research scholars, industrialists, etc. with their research work and provide ease in searching through the findings of the current research work in CRC. It also helps to identify the diagnostic, prognostic, and predictive biomarkers. Thus, it provides a conceptual knowledge of nano-based medicine for the management of CRC. 

## 13. Conclusions

Many people around the world are still affected by CRC. mCRC management remains a challenge despite screening recommendations and advancements in treatment options. A variety of genetic and epigenetic factors contribute to the heterogeneity of CRC. There is also much variability in the tumour microenvironment in CRC, making it difficult to find therapeutic targets for people with metastatic CRC. The heterogeneity of tumours within and across primary and metastatic sites can be extremely diverse. This could be a contributing factor to a treatment failure when a patient develops a resistance to targeted medicines. Since the effectiveness of more recent focused treatments depends on a good understanding of the patient’s previous response, a tumour specimen analysis is also required for understanding the underlying illness mechanism and/or tumour resistance traits. As our understanding of the colorectal carcinogenesis molecular landscape expands, new molecular biomarkers with prognostic and predictive information are being identified. For the real-time examination of tumour clonal development, medication response, the existence of minimum residual illness, and acquired resistance, liquid biopsies are a potential approach. CRC patients may benefit from real-time molecular categorisation and customised treatment using non-invasive biomarkers. With a better understanding of the epigenetic regulatory mechanisms, including cancer-specific epigenetic changes, prospective clinical implications as diagnostics or therapeutic targets in colorectal cancer can now be explored more thoroughly. 

The diagnostic efficacy of these biomarkers in early-stage CRC screening, prognostication, or treatment response prediction has yet to be improved, and most of these biomarkers have not yet been validated in large independent patient cohorts. A significant step towards individualised medicine should, in our opinion, include the consideration of non-cancer-related aspects such as nutrition and lifestyle. The existing methods in the cancer management sector have been rapidly replaced by new ones that offer greater diagnostic capabilities, increased sensitivity for tumour staging, and better therapeutic approaches. There are various advantages to using nanotechnology in colon cancer treatment. We can see early indications of nanotechnology’s value in evaluating colorectal cancer imaging, medicines, and ablation methods. The first measures have already been taken to improve these areas. There should be no doubt that nanotechnology-based techniques have the potential to treat cancers other than CRC, even if this opinion has concentrated on CRC as a particularly attractive target for early application.

## Figures and Tables

**Table 1:** An assessment of the MSI-H, EMAST, CIMP, and CIN CRC indices.

Pathophysiological Routes	Genomic Instability	Inflammation	Prognosis	Pathogenesis
CIN	Causes to the mutation and copy number variation; MSS; aneuploid	Tumour margin, lamina propria, and intraepithelial sites all have varying degrees of differentiation	Referent	Genetic changes that lead to heterozygosity loss
CIMP	Leading hypermethylation at DNA loci	Without hMLH1 hypermethylation: varied	Poor survival without hMLH1 hypermethylation	Without hMLH1 hypermethylation: unknown
MSI-H	Microsatellite instability (MSI) and diploid is appeared	Crohn’s-like around tumour (tumour margin)	Better survival; early stage	Target gene frameshift mutation; BRAF^V600E^
EMAST	Instability found in mostly at MSS and MSI-L, includes MSI-H	Tumour nests surrounding epithelial components have been linked to this condition.	Poor survival; later stage	Chromosome instability in combination with a frameshift mutation on a target gene

**Table 2:** Illustration of completed and ongoing clinical trials evidence for CRC.

Title	Location	Trial Identifier	Phase and Status
Metastatic Colorectal Cancer Database	Methodology, Biostatistics and Data Management Dijon, France	NCT04031625	Not applicable, Recruiting
Maintenance Therapy for Metastatic Colorectal Cancer After First-Line Treatment with Fruquintinib Plus Capecitabine Versus Bevacizumab Plus Capecitabine	Medical College of Zhejiang University Hangzhou, China	NCT04733963	Phase 2, Recruiting
CRC Patients having liver metastases are being treated with TAS-102 and radiation therapy	Massachusetts General Hospital, United States	NCT03223779	Phase 1, Recruiting
Anlotinib Combined With mXELIRI as Second-line Treatment of Advanced Colorectal Cancer	Guangdong Provincial Hospital of Chinese Medicine Guangzhou, China	NCT05035914	Phase 1, Recruiting
Do Colorectal Cancer Risk Estimates Affect Screening Behavior?	Stanford University School of Medicine Palo Alto, US	NCT03819920	Not Applicable, Completed
Study of Fruquintinib Efficacy and Safety in Patients with 3+ Line Colorectal Cancer (FRESCO) in Phase III	Hutchison Medi Pharma Investigational Site Hefei, Anhui, China	NCT02314819	Phase 3, Completed
Gut Microbiome Dynamics in Metastasized or Irresectable Colorectal Cancer	Wilhelmina Ziekenhuis Assen, Netherlands	NCT03941080	Not Applicable, Recruiting
Dabrafenib + Trametinib + PDR001 In Colorectal Cancer	Massachusetts General Hospital Cancer Center	NCT03668431	Phase 2, Recruiting
Colorectal Cancer Research Consortium (NCRCC) Study: National Colorectal Cancer Research Consortium	Zhejiang University College of Medicine Hangzhou, China	NCT04074538	Not Applicable, Recruiting
A Translational Study Examines the Impact of the MET Oncogene in Human Colorectal Cancer	Fondazione del Piemonte per l’Oncologia Candiolo, Italy	NCT02238821	Not Applicable, Completed
The Use of a Patient Navigator to Increase Colorectal Cancer Screening Uptake	Group Health Centre Sault Ste. Marie, Canada	NCT01506687	Phase 3, Completed
Regorafenib in the Treatment of Patients with Metastatic Colorectal Cancer: Real-World Effectiveness	Henan Cancer Hospital Zhengzhou, China	NCT05023720	Not Applicable, Recruiting
Investigating the effects on colorectal cancer patients of a walking programme	National Taiwan University Hospital Taipei, Taiwan	NCT01595256	Not Applicable, Completed
Metabolomics-Based Detection of Colorectal Cancer	Indiana University Cancer Center Indianapolis, US	NCT00507598	Not Applicable, Completed
Exploration into how often patients with colorectal cancer who are receiving irinotecan-based therapy experience nausea and vomiting	Caritas St. Elizabeth Medical Center Brighton, US	NCT00713128	Not Applicable, Completed
A Colorectal Cancer Screening Decision Aid for American Indians That Is Culturally Adapted	Robeson Health Care Corporation Lumberton, US	NCT03569761	Not Applicable, Completed
Vaccination Against MSI Colorectal Cancer	Krankenhaus Nordwest Frankfurt/Main, Germany	NCT01461148	Phase 2, Completed
Fecal Occult Blood Tests for Colorectal Cancer Screening: A Comparison of the Different Methods	Qilu Hospital Jinan, China	NCT04454099	Not Applicable, Completed
Concern for Positron Emission Tomography (PET) in Colorectal Cancer Stage II and III Follow-Up	Cancérologie et Hépato-Gastro-Entérologie Bordeaux, France	NCT00199654	Phase 3, Completed
Relapsed/refractory Colorectal Cancer Patients Receiving IMMU-130 Trial	Memorial Sloan-Kettering Cancer Center New York, US	NCT01270698	Phase 1, Completed
